# Web-Based Learning for Emergency Airway Management in Anesthesia Residency Training

**DOI:** 10.1155/2015/971406

**Published:** 2015-12-16

**Authors:** Ada Hindle, Ji Cheng, Lehana Thabane, Anne Wong

**Affiliations:** ^1^Department of Anesthesia, McMaster University, 1280 Main Street West, Hamilton, ON, Canada L8S 4K1; ^2^Biostatistics Unit, St. Joseph's Healthcare Hamilton, McMaster University, 50 Charlton East, Hamilton, ON, Canada L8N 4A6; ^3^Department of Clinical Epidemiology and Biostatistics and Department of Pediatrics, McMaster University, 1280 Main Street West, Hamilton, ON, Canada L8S 4K1

## Abstract

*Introduction*. Web-based learning (WBL) is increasingly used in medical education; however, residency training programs often lack guidance on its implementation. We describe how the use of feasibility studies can guide the use of WBL in anesthesia residency training.* Methods*. Two case-based WBL emergency airway management modules were developed for self-directed use by anesthesia residents. The feasibility of using this educational modality was assessed using a single cohort pretest/posttest design. Outcome measures included user recruitment and retention rate, perceptions of educational value, and knowledge improvement. The differences between pre- and postmodule test scores and survey Likert scores were analysed using the paired *t* test.* Results*. Recruitment and retention rates were 90% and 65%, respectively. User-friendliness of the modules was rated highly. There was a significant improvement in perceptions of the value of WBL in the postsurvey. There was a significant knowledge improvement of 29% in the postmodule test.* Conclusions*. Feasibility studies can help guide appropriate use of WBL in curricula. While our study supported the potential feasibility of emergency airway management modules for training, collaboration with other anesthesia residency programs may enable more efficient development, implementation, and evaluation of this resource-intensive modality in anesthesia education and practice.

## 1. Introduction

Web-based learning (WBL) can be defined as the “usage of computers and networks in education,” including learning management systems, online tutorials, discussion forums, and simulation [1, 2]. WBL has become increasingly used in medical education; however, it is important to understand how to best design and implement it as an educational modality [3–20].

Difficult and emergency airway management is an essential skill to acquire during anesthesia residency training. WBL may complement preexisting curricula and address gaps in clinical training in this area. Advantages of WBL include flexibility and access, enhancement of other educational modalities, ease for content updating, and appeal for the current “millennial learners” [21, 22].

While learning theories can guide the pedagogical use of WBL, major barriers to implementation in residency include time, cost, and technical expertise requirements as well as inadequate learner and faculty uptake [20, 21]. Therefore, WBL is effective only if successfully integrated in curricula and consistently used by learners [23]. Given the comparable educational outcomes of WBL and traditional methods [18, 19, 21], it is crucial to assess the feasibility [24] of WBL implementation in order to balance expense and educational benefits, especially for departments with limited resources.

The WBL literature provides little guidance on how to assess the feasibility of implementing a new teaching modality. Therefore, the purpose of this paper is to describe how we developed WBL modules for emergency airway training and explored their feasibility for implementation into our anesthesia residency curriculum at McMaster University, Canada. Feasibility is assessed by examining the recruitment and retention rate, user perceptions, and knowledge improvement. We hope our experience will inform other training programs that are considering incorporating WBL into their curricula.

## 2. Materials and Methods

### 2.1. Module Design and Development

We conducted a literature review and consulted an educational technology instructional designer in order to develop the modules according to principles of effective instructional web design [21–23]. Based on active learning theories, aspects of WBL design that improve its educational efficacy include interactivity, practice exercises, feedback, and repetition [2, 18, 21, 23, 25]. Embedded case-based, self-assessment questions have also been shown to improve learning outcomes [26].

We selected two common emergency airway topics (burn and facial trauma) and developed them into two separate case-based airway modules for self-directed online use. Background information was provided in each patient scenario along with relevant images and diagrams. Embedded multiple choice questions allowed the participant to interactively make clinical management decisions throughout the modules. After selecting an answer to a question, the user received immediate feedback with a detailed explanation.

We developed the study objectives and content based on information from the Airway Evaluation and Management Guidelines of the National Curriculum for Canadian Anesthesia Residency [27], anesthesia textbooks [28–30], and expert opinion papers [31–35]. The content of the modules was peer-reviewed by several faculty anesthesiologists and revised for clarity and appropriateness.

The study instruments included pre- and postmodule survey and knowledge test. The pre- and postmodule surveys (supplemental content in Supplementary Material available online at http://dx.doi.org/10.1155/2015/971406) assessed the participants' perceptions of the value and user-friendliness of WBL and other educational modalities. The pre- and postmodule knowledge tests assessed the module content. The modules, surveys, and tests together formed the Training for Emergency Airway Management (TEAM) course, which was launched on Avenue to Learn (a computer-based McMaster learning management system).

After logging into Avenue to Learn, residents were directed to complete TEAM in the following order: premodule knowledge test and survey, the two airway modules, and finally the postmodule knowledge test and survey. Residents had five weeks to complete TEAM. Further details of how the modules and study instruments were created are outlined in the supplemental content. The total time for the development, design, testing, and launch of TEAM was 165 hours.

### 2.2. Feasibility Study Procedures

This study was approved by the McMaster University Research Ethics Board. We used a single cohort pretest/posttest design where participants served as their own control [36]. Because TEAM was developed for junior residents, only the current year's cohort of anesthesia residents in postgraduate years (PGY) 1–4 (total study population = 29) were invited to participate.

### 2.3. Data Collection

Data from the participant responses on Avenue to Learn were exported to Excel data files, deidentified, and assigned individual codes by our Research Coordinator before analysis by the investigators.

### 2.4. Outcome Measures

The feasibility of incorporating WBL into the curriculum was assessed by the recruitment rate (% of participants/total study population) and retention rate (% of participants who successfully completed entire TEAM/total participants), user perceptions (reported as mean Likert scores), and knowledge improvement (measured by comparing pre- and postmodule knowledge test scores).

This WBL program would be considered feasible [24] if the following predefined criteria were met: (1) “definitely feasible” if recruitment rate and retention rate were at least 80%, (2) “possibly feasible” if recruitment rate and retention rate were between 60 and 79%, (3) mean user perception ratings of at least 5 on a 7-point Likert scale, (4) increased postmodule Likert scores of perceptions of the educational value of WBL, and (5) significant knowledge improvement between the pre-and postmodule test scores.

### 2.5. Statistical Analysis

Pre- and postmodule test scores and survey Likert scores were summarized as mean and standard deviation (SD). The differences between post- and premodule test scores and survey Likert scores were assessed using two-sided paired *t* tests. The results were reported as mean differences, with corresponding 95% confidence interval and associated *p* values. We set the level of significance at alpha = 0.05 and did not adjust this for multiple comparisons since these were primarily exploratory. Further exploratory analyses using regression were performed to assess associations between residency year and outcomes. All analyses were conducted using STATA 10.1 (College Station, TX).

## 3. Results

### 3.1. Recruitment and Retention

**Figure 1:**
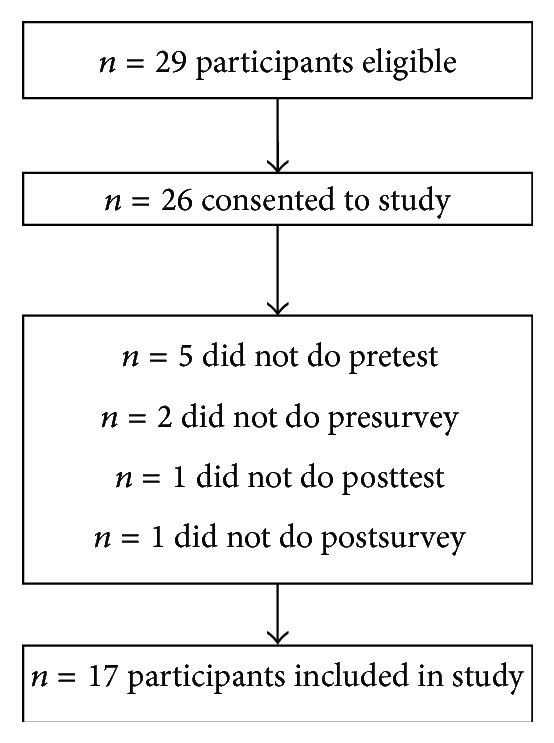
Flow diagram of residents' participation.

Twenty-six out of a total of 29 eligible anesthesia residents consented to the study, for a recruitment rate of 90% (Table 1). Eighteen of the 26 residents completed both the pre- and posttests, but one of these residents failed to complete the postsurvey (Figure 1). Therefore, the retention rate was 65% based on those 17 residents who completed all the pre- and postmodule tests and surveys. Incomplete data from the other participants were not included in the analysis.

### 3.2. User Perceptions

User perceptions were assessed by questions relating to user-friendliness and educational value of WBL, using a 7-point Likert scale. User-friendliness was perceived to be high, as measured by “ease of use” (mean score: 6.76, SD: 1.15), “interpretability” (mean score: 6.5, SD: 1.26), and “visual aid” (mean score: 5.35, SD: 2).

**Figure 2:**
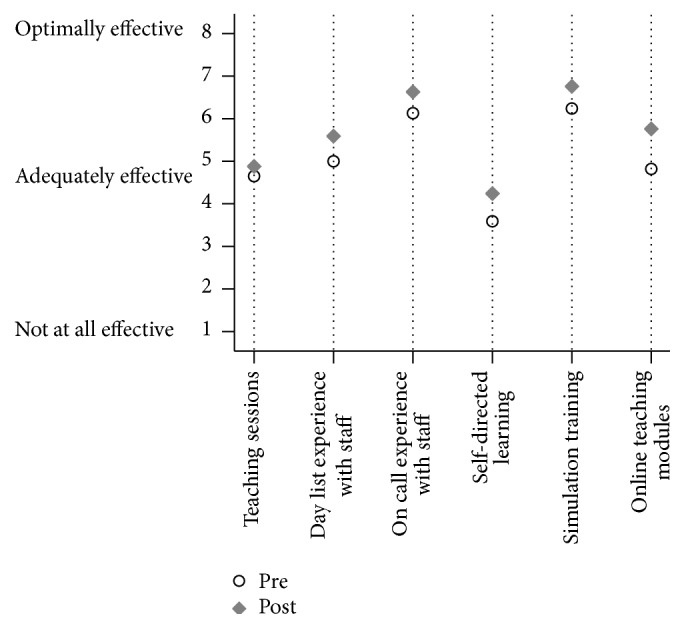
Participant perception of teaching modality value in premodule versus postmodule survey comparison.

With respect to perceptions of educational value, in the pre-and postmodule survey, residents ranked “simulation” and “on call experience with faculty member” as the first and second preferred learning methods, respectively, for learning to manage emergency airways (Figure 2). WBL was ranked in the middle with a mean value of 4.82 (SD: 1.55). Self-directed learning was ranked the lowest at 3.59 (SD: 1.37). After modules, both of these modalities increased significantly in perceived value with mean of 4.24 (SD: 1.25) (95% CI of 0.14 to 1.16, *p* = 0.017) and 5.76 (SD: 1.39) (95% CI of 0.41 to 1.47, *p* = 0.002), respectively, suggesting a positive impact on perceptions from using WBL.

### 3.3. Knowledge Improvement

Knowledge improvement was measured by comparing the pre- and postmodule knowledge scores. The mean knowledge test scores were calculated out of 14 questions, instead of the original 15, due to a reported broken image link on one of the questions. A comparison analysis revealed no statistical difference between pre- and postmodule test scores of 14 questions compared to 15. On average, residents scored a mean of 7.39 (SD: 1.97) out of 14 on the premodule test (Table 2). The postmodule test score was significantly higher, with a mean of 11.44 (SD: 1.72) (*p* < 0.001). This represented 29% improvement in the postknowledge test.

**Figure 3:**
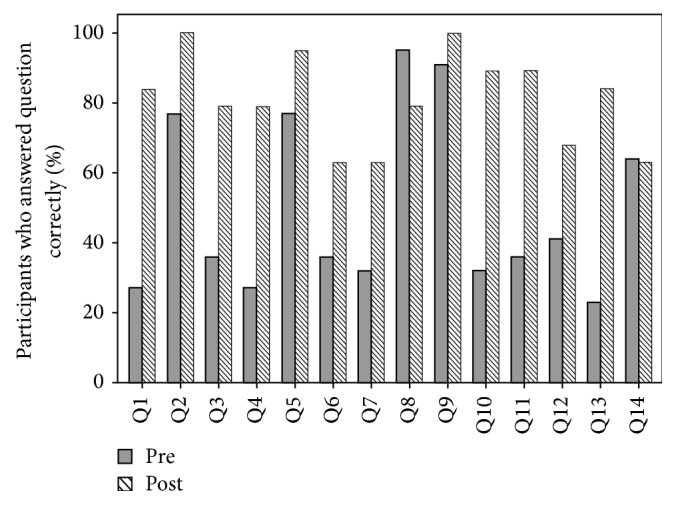
Individual question breakdown in pretest versus posttest comparison for percentage of participants who answered correctly.

Regarding each individual test question, a predictably higher percentage of participants answered the posttest version correctly compared to the pretest version, with the exception of two questions, possibly due to ambiguous wording or content (Figure 3). Overall, premodule test scores tended to increase slightly with the level of training, although there was no statistically significant difference in scores between junior and more senior residents.

## 4. Discussion

WBL is increasingly being used in anesthesia training. Bello et al. [9] demonstrated that the combination of online lecture slides and videos of difficult airway procedures resulted in an increase in postknowledge test scores and positive satisfaction scores. Soto et al. [17] paired didactic online lectures on anesthesia drug costs with “in-person” presentations to improve knowledge scores. Kopp and Smith [14] showed improved test scores in regional anesthesia with both case-based and traditional textbook style modules.

In their meta-analysis and systematic review, Cook et al. [18, 19] concluded that WBL is effective compared with no intervention and is at least as effective as traditional educational interventions. Therefore, given these findings, research should focus on examining the appropriate conditions for effective WBL implementation and use [18, 19]. In order to examine the usefulness of WBL in enhancing emergency airway management training in our curriculum and guide further development, we assessed the feasibility of implementing WBL modules into the anesthesia curriculum with respect to user uptake, perceptions, and educational value.

According to our feasibility criteria, the findings suggest that implementing interactive WBL emergency airway management modules in our anesthesia curriculum is “possibly feasible” as demonstrated by the recruitment and retention rates, user satisfaction, and evidence of educational value. The high recruitment rate of 90% is promising, suggesting that almost all of the residents were interested in WBL. However, the lower retention rate of 65% is problematic, pointing to a need to further examine contributing factors, such as technical issues, complexity or length of the modules, and/or the study measurement tools.

The user-friendliness of the modules in terms of ease of use and interpretability were highly rated. The lower rating on the use of visual aids might have been improved with more images or other multimedia. With respect to perceptions of educational value, resident “buy-in” for the use of WBL is further affirmed by a significantly increased mean Likert score and ranking of the postmodule survey compared with the premodule survey (4.8, SD: 1.55, versus 5.76, SD: 1.39). The educational value and construct validity of these modules are also supported by a significant overall improvement in postknowledge test scores.

Of all the educational modalities, “self-directed learning” received the lowest rating of 3.59 (SD: 1.37) and although it did significantly improve to 4.24 (SD: 1.25), it still remained low. The low ratings could have resulted from varying interpretations of what the term meant (as we did not clearly define it). Furthermore, it was asked separately from the WBL question; therefore residents may not have made the connection between self-directed learning and WBL. It is also possible that, in the critical area of emergency airway management, residents preferred to receive more instructor guidance than would be provided by self-directed learning. This finding warrants further investigation.

On call experience with faculty and simulation training were valued the highest both before and after survey. This finding is consistent with studies which show that learners often seek experiential knowledge around the context of patient encounters [37, 38]. Similar to other studies [39–42], our findings suggest that WBL is regarded as a valued supplement but not replacement for current experiential educational modalities.

The results of our feasibility study as well as our systematic evidence-informed approach [43–45] to the development of these modules help inform our decision to implement WBL as a potentially efficacious modality that will enhance anesthesia residency training. Based on these outcomes, future development would include improving the technical aspects of WBL design, addressing the limitations of the learning management system, examining issues associated with retention, and incorporating facilitated online discussions.

There were several outcomes that we were unable to report due to limitations in the learning management system. The software program did not allow comments to be collected from participants, reporting on the length of time spent on the different components or identifying whether “incorrect” questions were truly incorrect or simply not attempted at all. While knowledge improved after the modules, longer term knowledge retention needs to be assessed. We did not enable any online forums in order to maintain the participant's anonymity; however, this should be considered as a venue for feedback in order to improve the modules. We cannot generalize our specific results to other programs; nevertheless, our approach to developing WBL modules as well as assessing feasibility of incorporating WBL is helpful to inform other programs that are considering incorporating WBL in their curricula.

## 5. Conclusion

In conclusion, our study shows how we can assess whether WBL is a feasible and potentially efficacious educational modality for implementation into an anesthesia training curriculum. Although the initial time and resource commitments were substantial, our findings suggest the possible feasibility of using WBL modules to enhance the clinical training in difficult and emergency airway management. It would be important to consider collaborating with other anesthesia training programs in order to pool resources and more efficiently develop, implement, and further evaluate this educational modality for anesthesia education and practice.

## Supplementary Material

The supplemental material consists of the pre-module and post- module surveys as well as information on how the online modules were constructed and uploaded on the learning management system. Screen shots of the facial trauma module are included for further information.

## Figures and Tables

**Table 1:** Participants' demographics (*n* = 17).

Characteristics	*n* (%)
Gender	
Male	8 (47)
Female	9 (53)
Residency year	
PGY 1	6 (35)
PGY 2	6 (35)
PGY 3	2 (12)
PGY 4	3 (18)

PGY, postgraduate training year; *n*, sample size of participants that completed all components of TEAM.

**Table 2:** Pre- and postmodule comparisons of tests and surveys.

	Premodule	Postmodule	Difference		
	Mean (SD)	Mean (SD)	Post-pre	(95% CI)	*p* value
Module survey					
Teaching sessions	4.65 (1.62)	4.88 (1.22)	0.24	(−0.63, 1.10)	0.512
Day list experience with staff	5 (1.94)	5.59 (1.37)	0.59	(−0.44, −1.62)	0.243
On call experience with staff	6.13 (2.19)	6.63 (1.15)	0.50	(−0.58, 1.58)	0.341
Self-directed learning	3.59 (1.37)	4.24 (1.25)	0.65	(0.14, 1.16)	0.017
Simulation training	6.24 (1.82)	6.76 (1.20)	0.53	(−0.05, 1.11)	0.070
Online teaching modules	4.82 (1.55)	5.76 (1.39)	0.94	(0.41, 1.47)	0.002
Test					
Test score (out of 14)	7.39 (1.97)	11.44 (1.72)	4.06	(3.15, 4.97)	<0.001
Test percentage	52.78 (13.93)	81.94 (12.35)	29.17	(22.73, 35.61)	<0.001

95% CI, 95% confidence interval. Results are expressed as means with standard deviation (SD).

Two-sided paired *t* tests for differences.

## Abbreviations

WBL: Web-Based Learning
TEAM: Training for Emergency Airway Management
PGY: Postgraduate year
SD: Standard deviation.
