# Supplementary material for: Web-Based Learning for Emergency Airway Management in Anesthesia Residency Training
Source: Anesthesiol Res Pract. 2015 Dec 16;2015:971406. doi: 10.1155/2015/971406 (PMC4695649; doi:10.1155/2015/971406)
Supplement: Supplementary file 1 — The supplemental material consists of the pre-module and post- module surveys as well as information on how the online modules were constructed and uploaded on the learning management system. Screen shots of the facial trauma module are included for further information. [file 971406.f1.pdf]

SUPPLEMENTAL CONTENT

1. **Pre-module survey**
2. **Post-module survey**
3. **Supplemental information on construction of modules, with accompanying figures i to iii .**

<u>Pre-module Survey</u>

1) Please rate the effectiveness of the following strategies as preparation to manage emergency airways while on call:

a) Teaching sessions, such as Academic day

1	2	3	4	5	6	7
Not at all			Adequate			Optimal

b) Day list experience with faculty

1	2	3	4	5	6	7
Not at all			Adequate			Optimal

c) On call experience with faculty

1	2	3	4	5	6	7
Not at all			Adequate			Optimal

d) Self-directed learning

1	2	3	4	5	6	7
Not at all			Adequate			Optimal

e) Simulation training

1	2	3	4	5	6	7

Not at all	Adequate	Optimal

f) Online teaching modules

1	2	3	4	5	6	7
Not at all			Adequate			Optimal

g) Other: _____________

1	2	3	4	5	6	7
Not at all			Adequate			Optimal

Post- Module Survey

1) Please rate the following with respect to the module:

a) Ease of use

1	2	3	4	5	6	7
Not at all			Adequate			Optimal

b) Interpretability of cases and questions

1	2	3	4	5	6	7
Not at all			Adequate			Optimal

c) Visual aid

1	2	3	4	5	6	7
Not at all			Adequate			Optimal

2) Based on the time spent on these cases, how many cases would you realistically do online as preparation for managing emergency airway management:

a) 1-2 more cases per year

b) 3-5 more cases per year

c) 6-10 more cases per year

d) 11 + more cases per year

3) Please rate the effectiveness of the following strategies as preparation to manage emergency airways:

a) Teaching sessions, such as Academic day

1	2	3	4	5	6	7
Not at all			Adequate			Optimal

b) Day list experience with faculty

1	2	3	4	5	6	7
Not at all			Adequate			Optimal

c) On call experience with faculty

1	2	3	4	5	6	7
Not at all			Adequate			Optimal

d) Self-directed learning

1	2	3	4	5	6	7
Not at all			Adequate			Optimal

e) Simulation training

1	2	3	4	5	6	7
Not at all			Adequate			Optimal

f) Online teaching modules

1	2	3	4	5	6	7
Not at all			Adequate			Optimal

g) Other: ______________

1	2	3	4	5	6	7
Not at all			Adequate			Optimal

<u>Supplemental information on construction of modules</u>

The modules were initially constructed in text format using PowerPoint and then built as interactive WBL modules with Articulate Storyline® software, with the help of an instructional designer at McMaster University.

The modules were uploaded by the instructional designer to “Avenue to Learn”, an online learning management system used at McMaster University. The pre- and post- module surveys and knowledge tests were then created in Avenue to Learn.

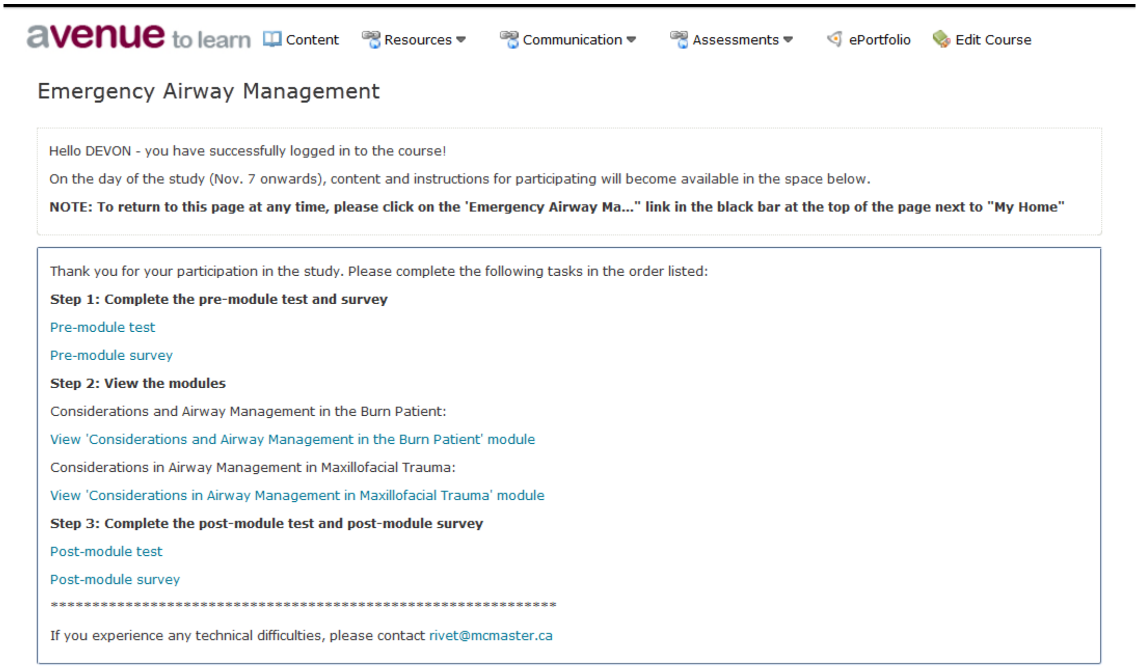

fig i. Emergency Airway Management course menu from Avenue to Learn® learning management system.

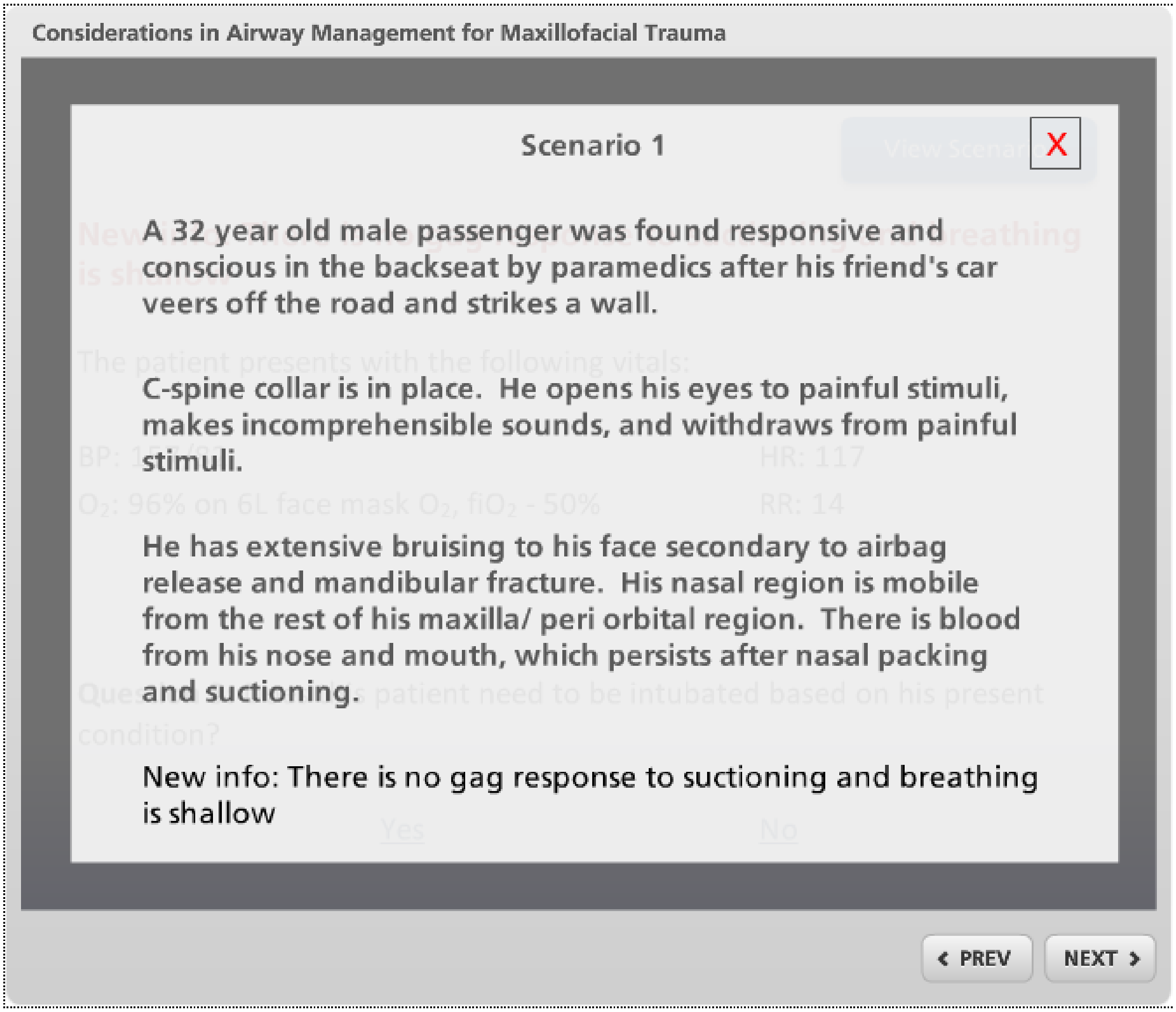

fig ii. Screenshot of case information for facial trauma module.

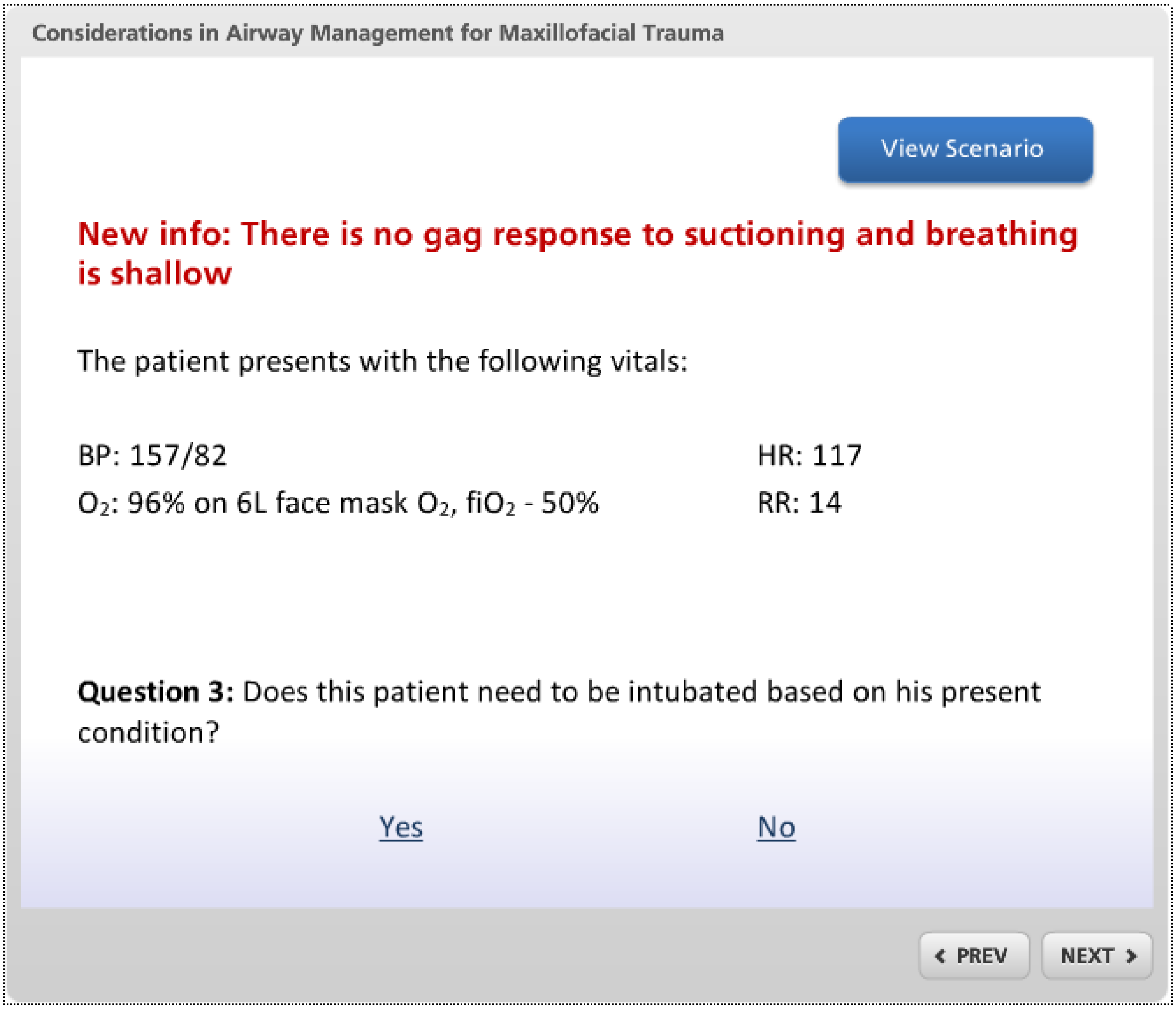

fig iii. Screenshot of questions for facial trauma module.
